# Strategies for repair of white matter: influence of osmolarity and microglia on proliferation and apoptosis of oligodendrocyte precursor cells in different basal culture media

**DOI:** 10.3389/fncel.2013.00277

**Published:** 2013-12-26

**Authors:** Karolina Kleinsimlinghaus, Romy Marx, Meray Serdar, Ivo Bendix, Irmgard D. Dietzel

**Affiliations:** ^1^Department of Biochemistry II, Ruhr University BochumBochum, Germany; ^2^Department of Pediatrics I, Neonatology, University Hospital EssenEssen, Germany

**Keywords:** oligodendrocyte progenitors, proliferation, osmolarity, microglia, culture medium, cytokines

## Abstract

The aim of the present study has been to obtain high yields of oligodendrocyte precursor cells (OPCs) in culture. This is a first step in facilitation of myelin repair. We show that, in addition to factors, known to promote proliferation, such as basic fibroblast growth factor (FGF-2) and platelet derived growth factor (PDGF) the choice of the basal medium exerts a significant influence on the yield of OPCs in cultures from newborn rats. During a culture period of up to 9 days we observed larger numbers of surviving cells in Dulbecco's Modified Eagle Medium (DMEM), and Roswell Park Memorial Institute Medium (RPMI) compared with Neurobasal Medium (NB). A larger number of A2B5-positive OPCs was found after 6 days in RPMI based media compared with NB. The percentage of bromodeoxyuridine (BrdU)-positive cells was largest in cultures maintained in DMEM and RPMI. The percentage of caspase-3 positive cells was largest in NB, suggesting that this medium inhibits OPC proliferation and favors apoptosis. A difference between NB and DMEM as well as RPMI is the reduced Na^+^-content. The addition of equiosmolar supplements of mannitol or NaCl to NB medium rescued the BrdU-incorporation rate. This suggested that the osmolarity influences the proliferation of OPCs. Plating density as well as residual microglia influence OPC survival, BrdU incorporation, and caspase-3 expression. We found, that high density cultures secrete factors that inhibit BrdU incorporation whereas the presence of additional microglia induces an increase in caspase-3 positive cells, indicative of enhanced apoptosis. An enhanced number of microglia could thus also explain the stronger inhibition of OPC differentiation observed in high density cultures in response to treatment with the cytokines TNF-α and IFN-γ. We conclude that a maximal yield of OPCs is obtained in a medium of an osmolarity higher than 280 mOsm plated at a relatively low density in the presence of as little microglia as technically achievable.

## Introduction

Cell cultures are used extensively to produce cell populations for tissue repair, and also for the investigation of the properties and interactions of populations of specific cells. Earlier cultures needed growth factors contained in fetal calf serum to allow cells to survive, but later serum free media were developed for cell growth under defined conditions. A first serum-replacing medium supplement contained five principal factors (insulin, transferrin, progesterone, selenium, and putrescine at optimized concentrations, termed N2). It was developed by Bottenstein and Sato for culturing B104 neuroblastoma cells (Bottenstein and Sato, 1979). Romijn et al. developed a more elaborate medium containing additional ingredients, including hormones and essential fatty acids (Romijn et al., 1984) which was then further refined to a medium called B18 by Brewer and Cotman to sustain the survival of neurons (Brewer and Cotman, 1989). Later further changes in the supplement were made (B27) and a new basal medium, providing the necessary electrolytes, amino acids, and vitamins (termed Neurobasal -NB) was developed to optimize maintenance of hippocampal neurons at a low background of astrocytes (Brewer et al., 1993). This medium has been widely used for culturing various cell types, as evident from more than 90 citations per year in the last decade. Problems with the quality of the commercially available supplements have recently led to the development of a slightly modified formulation, which contains 21 ingredients (NS21) (Chen et al., 2008).

Apart from culturing neurons the B27/NB medium can also be used to culture oligodendrocytes. For example, it has been used to investigate effects of inflammatory mediators, such as tumor necrosis factor-α (TNF-α) and interferon-γ (IFN-γ) on oligodendrocyte precursor cell (OPC) survival, differentiation, and ion channel expression in culture (Feldhaus et al., 2004a; Mann et al., 2008). A methodological study comparing the viability of OPC cultures in B27/NB medium and N1/DMEM medium provided evidence, that the B27/NB medium was significantly more effective in maintaining viable cells and in supporting oligodendrocyte proliferation than the combination N1/DMEM (Yang et al., 2005). The composition of the basal medium as well as the different supplements could have led to the higher success rate of the B27/NB combination. To study the influence of the basal culture medium on oligodendrocyte lineage cells we here systematically investigated the yield of surviving cells, their bromodeoxyuridine (BrdU) incorporation as measure for cell proliferation as well as caspase-3 immunofluorescence as a measure for the percentage of preapoptotic cells after culturing cells under three conditions differing only in the choice of the basal medium, [Dulbecco's Modified Eagle Medium (DMEM), Roswell Park Memorial Institute medium (RPMI), and Neurobasal Medium (NB)].

Furthermore, cultures obtained by differential adhesion contain microglia (Hewett et al., 1999). Microglia may secrete factors that are deleterious or supportive for the cultured oligodendrocytes. In order to investigate the influence of the remaining microglia we further investigated the impact on OPC proliferation and survival of adding surplus microglia to the culture.

This surplus of microglial cells and factors secreted from neighboring OPCs may also impact the responses of cultured OPCs to pharmacological treatments. This is demonstrated in a series of experiments investigating the response of OPC cultures of various densities to a standardized treatment with the cytokines TNF-α and IFN-γ.

## Methods

### Preparation of mixed glial cultures

The protocol for the preparation of glial cultures followed the general procedures described by McCarthy and de Vellis (1980) and Armstrong (1998) with some modifications. Postnatal 0–3 day-old Wistar Hannover rat pups were decapitated and the whole brain rostral of the cerebellum was removed and placed in a phosphate buffered saline (PBS)containing 137 mM NaCl, 2.7 mM KCl, 10.1 mM Na_2_HPO_4_, and 1.8 mM KH_2_PO_4_. After removal of inner and outer meninges the brains were passed successively through nylon meshes with pore sizes of 125 and 36 μm to remove neurons from the cell suspension. The dissociated cells were centrifuged [10 min, 900 rpm at room temperature (RT)] and the cell pellet resuspended in 5 ml glial mixed medium (GMM) composed of DMEM:Ham's F12 (1:1) supplemented with 10% heat inactivated fetal calf serum, 100 U/ml penicillin and 100 μg/ml streptomycin [all PAA, Germany)]. The obtained suspension was transferred into an uncoated T-75 flask, so that the cells of 1.5 brains were plated per flask. Ten milliliter of GMM were added, cultures were then allowed to grow at 37°C and 5% CO_2_ in a Haereus B5060 incubator (Hanau, Germany). Three to four days after plating the supernatant was centrifuged (5 min, 900 rpm), resuspended in fresh GMM and cells plated back onto the cell layer. Thereafter, the medium was replaced every 3–4 days without recycling cells from the supernatant.

### Isolation of oligodendrocyte progenitor cells

Cells were allowed to proliferate up to day 10–12 after preparation until the culture was composed of a confluent astrocyte layer adherent to the bottom of the flask with microglial cells and oligodendrocytes growing on top. The first step to obtain purified oligodendrocyte precursors was to remove the microglia by shaking the flasks (180 rpm) for 3 h on an orbital shaker ES-W (Kühner AG, Birsfelden, Switzerland). The supernatant, containing mostly microglia, was rejected, flasks were washed once with PBS, then fresh GMM was added and the flasks were shaken for another 18 h at 180 rpm to detach the oligodendrocytes from the astrocyte layer. The supernatant was centrifuged (5 min, 1700 rpm) and the cells contained in the pellet preplated in GMM (1 ml per flask) in non-coated petri dishes for 45 min at 37°C and 5% CO_2_ to remove remaining astrocytes from the suspension. The supernatant, containing mostly oligodendrocyte progenitors, was then centrifuged (5 min, 1000 rpm), the pellet resuspended in 1 ml GMM and cells counted in a Neubauer chamber. Cells were adjusted thereafter to densities of 5000, 20,000, and 80,000 cells per coverslip or Petri dish in GMM. Coverslips and Petri dishes were precoated with poly-L-lysine (5 μg/ml) to enhance attachment of the cells to the coverslips/Petri dishes. In the Petri dishes cells were first plated in sterile glass rings with the diameter of the coverslips (12 mm). After at least 1 h of adhering to the poly-L-lysine coated surfaces (at 37°C, 5% CO_2_) media were replaced by specialized media promoting proliferation.

Proliferation medium (PM) contained: Neurobasal (NB) (Invitrogen, Carlsbad, USA) medium, Roswell Park Memorial Institute (RPMI) (PAA, Cölbe, Germany), or Dulbecco's Modified Eagle's Medium (DMEM) (PAA, Cölbe, Germany) supplemented with 1xB27 (Sigma, Steinheim, Germany) without antioxidants, 100 U/ml penicillin (PAA), 100 μg/ml streptomycin (PAA, Cölbe, Germany), 10 ng/ml platelet derived growth factor (rHuPDGF-AA, Biomol, Hamburg, Germany, product no 50363), that inhibits differentiation and drives proliferation of O-2A glial progenitor cells into mature oligodendrocytes (Noble et al., 1988; Raff et al., 1988; Pringle et al., 1989) and10 ng/ml basic fibroblast growth factor (FGF-2, rHuFGF-basic, Biomol, Hamburg, Germany, product no 50361) that induces proliferation of OPCs (Bogler et al., 1990; McKinnon et al., 1991; Grinspan et al., 1993), see Bansal et al. (2002) for review. FGF-2 supplementations have been shown to saturate proliferation of O2-A precursor cells at 5–10 ng/ml (Besnard et al., 1989). For the present experiments we thus used the same concentrations of 10 ng/ml of PDFG and FGF-2 as employed by Back et al. (1998). Since in the experiments described here we attempted to study effects of basal media we did not study in more detail whether the yield of cells could be further optimized by varying the concentration of these growth factors or by adding further potential proliferation promoting factors, such as hepatocyte growth factor (HGF) (Yan and Rivkees, 2002), heregulin (HRG) (Canoll et al., 1996), insulin like growth factor-1 (IGF-1), (Zeger et al., 2007), or sonic hedgehog (Gao, 2006). In some experiments the osmolarity of the NB-medium (205 mOsm, as measured with a Knauer Type-ML osmometer) was increased to the osmolarity of the DMEM-medium (305 mOsm) by increasing the osmolarity of the NB medium by 100 mOsm by the addition of 52.5 mM NaCl or 100 mM mannitol.

After 3 days in PM cell culture media were exchanged to differentiation promoting media (DM). The media consisted of NB, RPMI, or DMEM supplemented with 100 U/ml penicillin (PAA), 100 μg/ml streptomycin (PAA, Cölbe, Germany), 45 nM triiodo-L-thyronine (T3) (Barres et al., 1994), 5 μM forskolin (Back et al., 1998), 10 ng/ml ciliary neurotrophic factor (CNTF) (Lopes-Cardozo et al., 1989), and 1xB27 without antioxidants (Sigma, Steinheim, Germany) per 50 ml.

Cytokine-treatment was performed 24 h after seeding for 48 h in PM/RPMI with recombinant rat IFN-γ (20 ng/ml) and TNF-α (10 ng/ml, both PeproTech, Hamburg, Germany). This cytokine mixture was chosen because in a preceding investigation on purified oligodendrocyte progenitors we had observed in accordance with Andrews et al. (1998), Melcangi et al. (2000), and Buntinx et al. (2004), that a combination TNF-α and IFN-γ is more potent in inducing apoptosis than either factor in separation.

### Isolation of microglia

Microglia were isolated from the mixed glia culture in the first shaking step during OPC isolation (see above). The supernatant containing the microglia was centrifuged (5 min, 1000 rpm at RT). The pellet was resuspended in 5 ml fresh GMM. Cells were plated in a T12 flask. Immediately before co-cultivation, the flasks were shaken for 5–10 min (180 rpm) to suspend the microglia.

### Co-cultivation of microglia and OPCs

The number of microglia in cultures with a seeding density of 80,000 cells was determined from an OX-42 staining. The evaluated density (2700 cells) of microglia was added to OPC-cultures with seeding densities of 5000 cells per coverslip/Petri dish. The glia cells were then co-cultivated for 3 days in PM. Control cultures were cultivated for 3 days in PM without additional microglia.

### Cultivation of OPCs with conditioned medium

Supernatants from 3 day old cultures with a seeding density of 80,000 cells per cover slip/petri dish were collected. Then fresh OPC-cultures seeded with a density of 5000 cells per coverslip/Petri dish were cultured for 3 days in either control PM or in the conditioned medium.

### Immunocytochemistry

Oligodendrocyte precursor cells (OPCs) were labeled with antibodies against A2B5 (Schnitzer and Schachner, 1982; Raff et al., 1983; Levi et al., 1987) and oligodendrocytes starting myelin production (OLs) were visualized by antibodies against myelin oligodendrocyte specific protein (MOSP). By immunoprecipitation anti MOSP antibodies have been shown to specifically bind to a 48 kDa membrane surface protein with a pI of 6.7 that is highly conserved in rodents, cats, monkeys, and humans (Dyer et al., 1991). Molecular masses of other myelin proteins such as myelin/oligodendrocyte glycoprotein (MOG) amount to 26–28 kDa, myelin basic protein (MBP) from 14 to 21.5 kDa, 2′,3′- cyclic nucleotide 3′- phosphodiesterase (CNPase) to 45 kDa, oligodendrocyte specific protein (OSP/claudin-11) to 22 kDa, and myelin associated glycoprotein (MAG) to 69 kDa (www.uniprot.org). MOSP expression correlates with increases in microtubular structures in oligodendrocytes and its initial expression occurs at the stage in development when oligodendrocyte processes have formed but membrane sheets have not yet been elaborated. This occurs shortly after galactocerebroside and sulfatide expression and 3 days before the expression of MOG (Mu and Dyer, 1994). In contrast to MOSP, OSP/claudin-11 expression, that is controversially discussed as a target for autoantibodies in multiple sclerosis (Aslam et al., 2010) occurs from the early progenitor stage and continues in mature oligodendrocytes (Bronstein et al., 2000). MOSP mediates signals that appear to increase the thickness and numbers of microtubular structures within oligodendrocyte membrane sheets and the preincubation of oligodendrocyte cultures with anti-MOSP antibodies leads to its redistribution from a uniform surface staining to lacy networks overlying microtubular structures that have CNPase colocalized along them. In contrast, incubation of cultures with antibodies against MOG induces an accumulation of MOG over internal domains of MBP, indicating that these sets of proteins play a distinct role in the cytoskeletal organization of oligodendrocytes (Dyer and Matthieu, 1994). A similar co-localization of MOSP and CNPase has later also been observed in the adult rhesus monkey brain where it was used as a marker for ongoing myelin formation (Sloane et al., 2003). Since MOSP belongs to the class of proteins involved in structuring the cytoskeleton of myelin (Dyer and Matthieu, 1994), it has later been used as marker for myelin producing oligodendrocytes (Gomes et al., 2003; Hoenicka et al., 2010; Iseki et al., 2011). We chose to use MOSP as marker to count the percentage of oligodendrocytes starting to produce myelin, since we had previously shown, that in cultures grown using the same protocol on the basis of NB-medium, as used here, MOSP stained cells with elaborate membrane processes appeared in parallel with an increase in MBP, MAG and CNPase [Figures 2B,C in Feldhaus et al. (2004a)] and since it showed a particularly clear specific staining of the membrane surfaces.

Mouse anti-A2B5 antibodies and anti-MOSP antibodies were obtained from Millipore [anti A2B5, clone A2B5-105 (MAB312), anti-oligodendrocytes, clone CE-1 (MAB328)], Alexa Fluor 488 labeled IgM and IgG anti-mouse antibodies and Alexa Fluor 594-labeled IgG anti-rabbit secondary antibodies were from Invitrogen, Carlsbad, CA. Primary and secondary antibodies were diluted in PBS to a concentration of 1:250 for first antibodies and 1:500–1:1000 for second antibodies. At the conclusion of each experiment the medium was removed, cells were then saturated with PBS with 3% goat serum for 30 min at RT. Afterwards cells were washed once with PBS, followed by the incubation with the first antibody for 1 h at RT. In the experiments shown in Figure 2, this was done in Krebs-Ringer (Krebs-Ringer-HEPES consisting of 115 mM NaCl, 5 mM KCl, 2 mM CaCl_2_, 1.2 mM MgSO_4_, 1.2 mM KH_2_PO_4_, 20 mM NaHCO_3_, 16 mM HEPES), whereas later A2B5 stainings were performed in PBS. To remove the excess of first antibody cells were washed once with PBS. After labeling the extracellular epitopes cells were then fixed with 4% paraformaldehyde (PFA) for 20 min. at RT. After washing cells twice with PBS, cells were incubated with secondary antibody for 1 h at RT during shaking. For counting progenitor cells one has to bear in mind that all antibodies staining progenitor cells, i.e., NG2, PDGFRα, and A2B5 do not specifically bind to bipolar progenitors but far beyond that point up to cells expressing various processes. Therefore progenitor cells referred to in the experiments investigating cytokine-effects shown in Figure 9 were identified morphologically. In preliminary experiments phase dark cells of bipolar shape bearing up to four unbranched processes had been identified to be 100% A2B5-positive. Using this definition the progenitor cell population counted in Figure 9 comprised a subpopulation of 70% of the A2B5-positive cells.

To determine the percentage of proliferating cells in culture, BrdU incorporation stainings were performed. For the BrdU staining cells were first incubated with BrdU (20 μM) for 20 h. After fixing the cells for 20 min with 4% PFA, they were washed with PBS. Cells were incubated with 37°C HCl (1M) for 1 h to uncover the DNA. Then cells were washed once with PBS. Afterwards cells were washed for 10 min with 100 mM borate buffer (15 mM sodium borate in distilled water, pH 8.3) to neutralize the remaining acid. Then the cells were incubated with a block buffer consisting of PBS-T (PBS with 0.1% Triton X-100) and 5% goat serum, followed by an overnight incubation with the primary antibody (*rat* anti-BrdU, Accurate chemicals OBT0030G) at 7°C used 1:750 in PBS. Thereafter cells were washed twice with PBS. The cells were then incubated with the secondary antibody (Alexa Fluor® 488 donkey anti-rat IgG, Invitrogen), that was used 1:750 in PBS. The duration of the incubation was 1.5 h at RT under shaking. Cells were then washed with PBS.

To determine the percentage of preapoptotic and apoptotic cells, caspase-3 and cleaved-caspase-3 stainings were performed. For the caspase-3 and the cleaved-caspase-3 staining cells were first fixed for 20 min with 4% PFA at RT, then submerged with hot citrate buffer (10 mM, pH 6 at 95°C) and incubated for 25 min at RT, followed by 3 washing steps, each 5 min, with PBS-T (PBS with 0.01% Triton X-100). Then the cells were incubated for 1 h at RT in a block buffer, consisting of PBS-T (PBS with 0.01% Triton-X) and 5% goat serum. Next cells were incubated with the first antibody (rabbit anti-Caspase-3, Cell Signaling, 9662) for the caspase 3 staining and with rabbit anti cleaved-caspase-3 antibody (New England Biolabs, 9661) overnight at 7°C. The antibodies were diluted 1:200 in block buffer. Cells were then washed three times with PBS, followed by the incubation with the second antibody [AlexaFluor® 594 goat anti-rabbit IgG, Invitrogen (A11012) diluted 1:500 in PBS] for 1 h at RT under shaking. Afterwards the cells were washed with PBS.

To identify DNA fragmentation in oligodendrocytes (*in vitro*) a TUNEL detection Kit (Roche 11684795910) was used. Cells, which had been fixed in 4% PFA for 20 min at RT were washed in PBS and incubated with permeabilization solution (0.1% Triton X-100 in 0.1% sodium-citrate) for 2 min on ice. After washing three times in PBS, the TUNEL mixture was prepared: The labeling solution (Fluorescein labeled nucleotides) was diluted in a ratio 1:4 and the enzyme solution (terminal desoxynucleotidyl transferase) in a ratio 1:50 in dilution buffer supplied by the Kit (11966006001). Each coverslip was incubated for 1 h at 37°C with 50 μl of this TUNEL mixture. Thereafter cells were washed with PBS and double stained with 4′,6 Diamidin-2-Phenylindol (DAPI 100 ng/ml 5 min at RT).

To calculate the amount of microglia in OPC cultures, OX-42 staining was performed. Cells were first fixed for 20 min with 4% PFA at RT, then incubated with block buffer, consisting of PBS-T (PBS with 0.1% Triton X-100) and 3% goat serum. Then cells were incubated with the primary antibody [mouse anti-rat CD11b IgG (anti-OX-42), Millipore, CBL1512Z], diluted 1:200 in PBS for 1 h at RT. Afterwards cells were washed with PBS, following the incubation with the secondary antibody (AlexaFluor® 488 goat anti-mouse IgG, Invitrogen, A11001), that was used 1:1000 in PBS. The incubation ensued 1 h at RT.

Finally nuclei of all cells were visualized by staining for 20 min at RT with DAPI (Sigma, Steinheim, Germany, diluted 1:2000 in earlier experiments) or Hoechst-33258 dye (10 ng/ml in PBS, Sigma-Aldrich, in later experiments). Fluorescence microphotographs were taken using a 20x objective on an Olympus (IX 51) microscope equipped with analysis^B^ software for earlier experiments and cellSens software for later experiments (Olympus) and a ColorView 12 camera. The higher light sensitivity of the more recently used microscope system could potentially account for overall larger numbers of BrdU positive and caspase-3 positive cells found in more recently performed experiments compared with earlier experiments. Results from an earlier experiment are shown for example in Figure 3. However, apart from overall larger percentages of stained cells counted more recently, all BrdU stainings qualitatively led to the same result, that cells cultured in NB show significantly lower BrdU incorporation rates. In contrast, changes in percentages of caspase-3 immunopositive cells got only significant in the more recent experiments, showing more than 5% positively stained cells.

**Figure 1:**
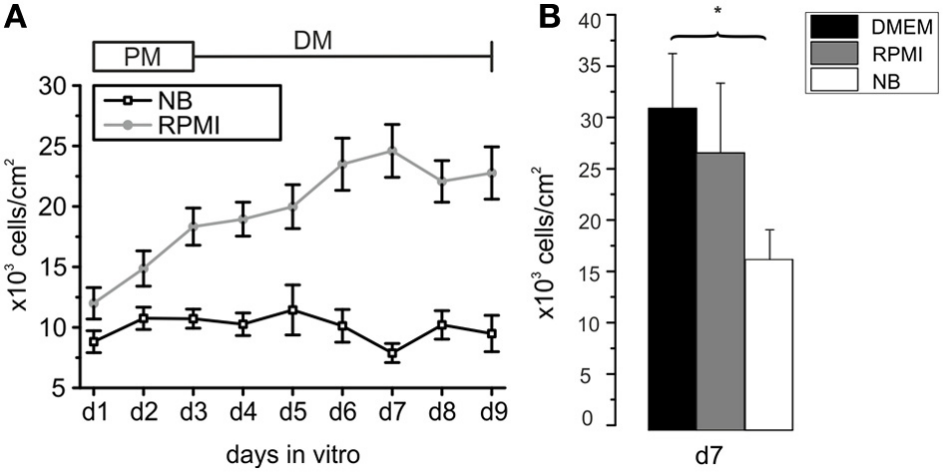
**Development of cell densities with time in culture in different basal media**. **(A)** Oligodendrocyte precursor cell cultures incubated from d1 to d3 in proliferation medium (PM) and for an additional 6 days in differentiation medium (DM) based on NB (black line) or RPMI medium (gray line) after purification. Each data point obtained by counting DAPI-stained nuclei from at least 8 photographed frames per preparation. Note the higher cell number from d1 to d9 [d3 to d9 (^*^p < 0.05)] in RPMI based medium compared with NB based medium (13 preparations in RPMI and 7 preparations in NB investigated). **(B)** Comparison of number of cells/cm^2^ surviving after 7 days in serum free media differing exclusively in the choice of the basal medium. Note that the highest rate of surviving cells was found in DMEM. (^*^p < 0.05, error bars indicate ± s.e.m.).

For cell counts 8–10 frames of randomly chosen, non-overlapping fields were photographed. Cell numbers were determined by counting Hoechst-33258 or DAPI stained nuclei in each frame. Numbers of cells on a coverslip or in a Petri dish were then calculated by determining the average cell number per frame and multiplied by the conversion factor between the area of the frame and the area of the coverslip. The percentage of BrdU and caspase positive cells was determined by divison of the number of the stained cells through the total number of the nuclei from morphologically identified oligodendrocyte lineage cells. Statistical analysis was performed using One-Way ANOVA with Tukey *post-hoc* test or paired Student's *t*-test if only two columns were compared. Results are given as mean ± standard error. Results shown in Figures 1, 5, 6 as significant using ANOVA showed even stronger significances when the single columns under treatment conditions were compared with controls using Student's *t*-test.

## Results

### Influence of basal media on proliferation and differentiation of purified OPCs

The first goal of this study was to investigate the impact of basal culture media on the number of OPCs surviving in culture, leaving the other supplements (B27 without antioxidants plus PDGF and FGF-2 in PM and forskolin, T3 and CNTF in differentiation medium) unchanged. Following an initial increase in cell number in NB-based PM the number of cells in the culture remained almost constant during the following 6 days in differentiation medium (Figure 1A). In contrast, cells grown for d1–d3 in RPMI-based PM and further 6 days in RPMI-based differentiation medium showed a continuous increase in cell number that had more than doubled after 9 days in culture. Every day *in vitro* from d1 to d9 the cell number was higher in RPMI based medium than in NB based medium, the difference reaching significant values from d3 on (^*^p < 0.005). In many studies DMEM is used as basal medium for oligodendrocyte cultures. We performed additional experiments in which we compared the outcome of sister cultures maintained for 3 days in PM and further 4 days in differentiation medium based on NB, RPMI as well as DMEM. The highest yield of cells was found in cultures maintained in DMEM, as shown in Figure 1B.

**Figure 2:**
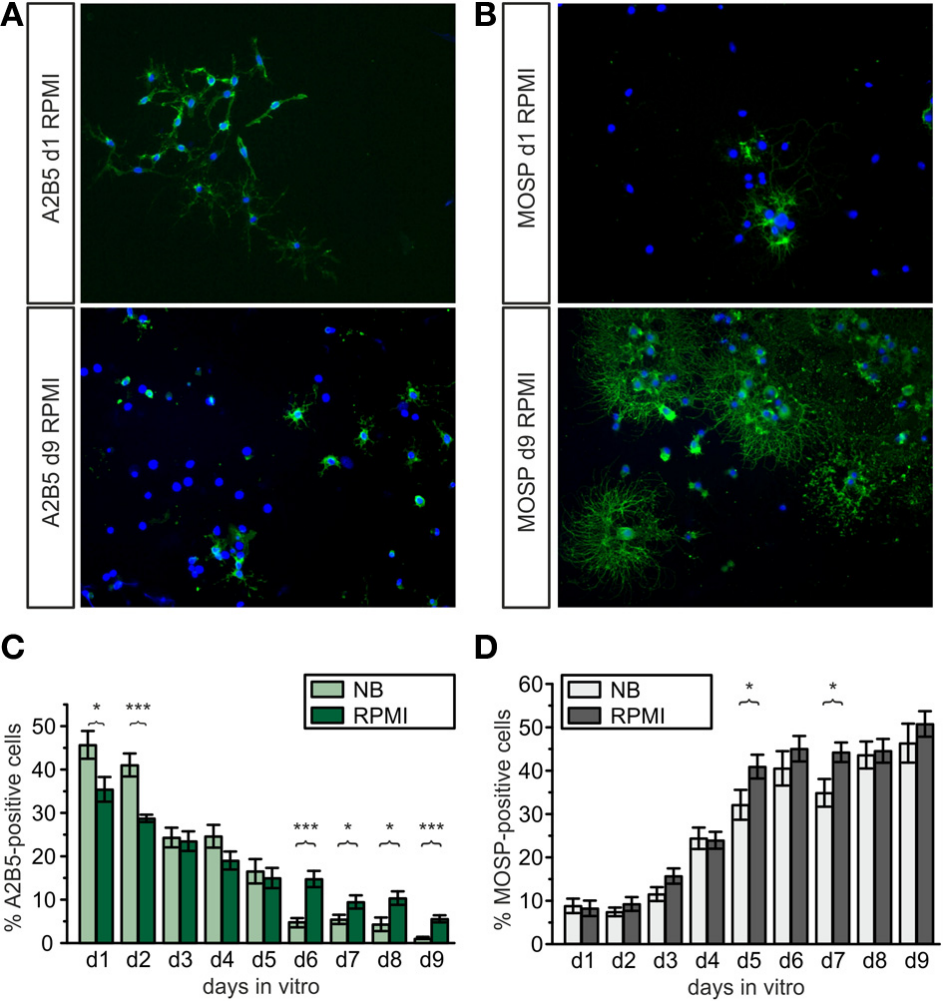
**Development of A2B5-positive progenitors and MOSP-positive oligodendrocytes in proliferation and differentiation media of different composition**. **(A,B)**: Examples for A2B5- and MOSP-immunopositive cells cultured in RPMI vs. NB and stained at d1 and d9 after isolation. **(A)** Immunostained pictures of A2B5-positive cells, showing approximately 35% A2B5-positive cells at d1 (top) and 5% at d9 (bottom). **(B)** Immunostained pictures (right) of approximately 5% MOSP-positive cells at d1 (top) and almost 50% at d9 (bottom). Nuclei counterstained with DAPI. **(C)** Direct comparison of percentage of A2B5-positive cells cultured in Neurobasal and RPMI based media, **(D)** direct comparison of MOSP-positive cells cultured in either Neurobasal or RPMI based media (^*^p < 0.05, ^***^p < 0.005; error bars indicate ± s.e.m., paired Student's *t*-test between pairs of bars).

To obtain further information how different basal culture media may influence the maturational stage of the oligodendrocyte lineage cells cultures on cover slips were stained with A2B5- and MOSP-antibodies (Figure 2) after growth in NB and RPMI based media. Earlier investigations have already shown, that the percentage of A2B5 positive OPCs progressively decreases while the number of MOSP-positive cells increases in parallel with increased levels of the myelin markers CNPase, myelin associated glycoprotein (MAG) and myelin basic protein (MBP) within 6 days in NB based differentiation medium [see Figure 2 in Feldhaus et al. (2004a)]. In the present experiments in both media the total number as well as the percentage of A2B5-positive cells decreased with progression of maturation of the OPCs (in NB from 46 to 1%, in RPMI from 35 to 6%, Figure 2C) and the total number as well as percentage of MOSP-positive cells increased (in NB from 9 to 46%, in RPMI from 8 to 51%) from d1 to d9 (see Figure 2D). Although in both media about 50% of the cells had differentiated to MOSP-positive cells after 6 days in differentiation medium, some differences emerged: Cells incubated in NB medium showed significantly higher percentages of A2B5-positive progenitor cells at d1 and d2. However, from d6 to d9 cells grown in RPMI-based medium displayed a significantly higher percentage of progenitor cells (Figure 2C). This observation is consistent with the finding shown in Figure 1, indicating that the total number of cells cultured in RPMI increased continuously. Hence this basal medium sustains a higher background of proliferating A2B5-positive progenitor cells.

### Effect of basal culture media on proliferation and apoptosis

**Figure 3:**
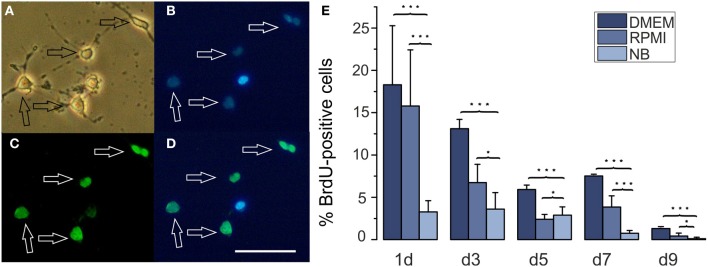
**Effects of different basal culture media on BrdU incorporation**. **(A–D)** Examples of a BrdU stained culture **(A)** phase contrast picture, **(B)** Hoechst 33258 staining of the same image, **(C)** BrdU stain, **(D)** merged pictures **(B)** and **(C)**, calibration bar represents 50 μm. Cells had been cultured in DMEM for 3 days. **(E)** Percentage of BrdU positive cells in cultures maintained for the days indicated below the columns in NB, DMEM as well as RPMI based media (^*^p < 0.05, ^***^p < 0.005; error bars indicate ± s.e.m.).

**Figure 4:**
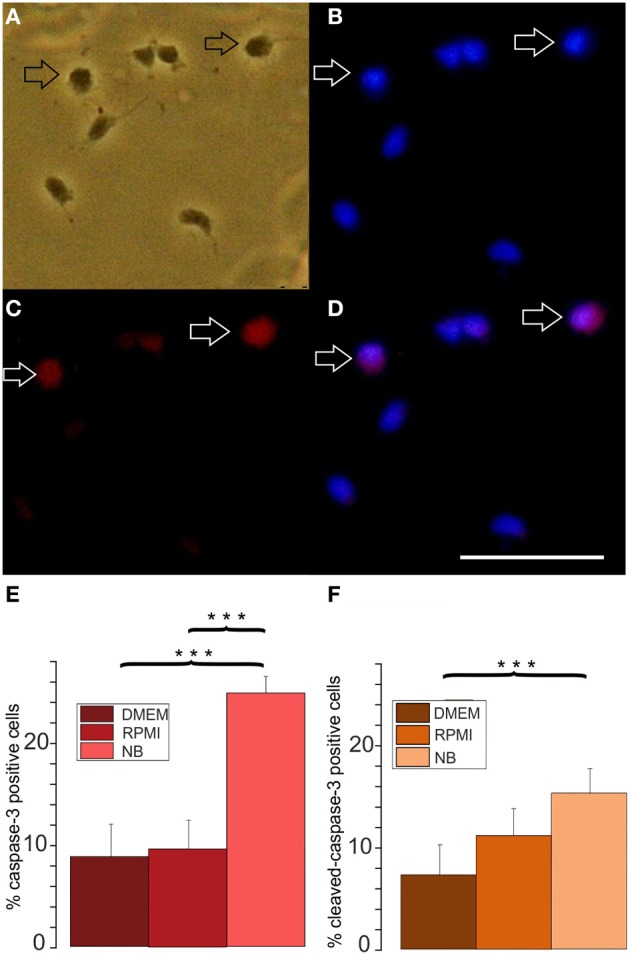
**Effects of different basal culture media on caspase-3 immunopositivity. (A–D)** Examples of a caspase-3 stained culture: **(A)** phase contrast picture, **(B)** Hoechst 33258 staining of the same image, **(C)** caspase-3 immunofluorescence, **(D)** merged pictures **(B)** and **(C)**, calibration bar indicates 50 μm. Cells stained after culture in proliferation medium based on DMEM for 3 days **(E)** percentage of caspase-3 positive cells and **(F)** cleaved caspase-3 positive cells after 3 days of culture in NB, DMEM as well as RPMI based media, (8 coverslips from 5 preparations evaluated, ^***^p < 0.005; error bars indicate ± s.e.m.).

A larger number of surviving cells and a larger number of remaining A2B5 positive cells in RPMI compared with NB could have resulted either from a decreased proliferation or an increased apoptosis in the later medium. In order to investigate whether one or both possibilities apply we tested, whether the percentage of OPCs showing BrdU incorporation as an indicator of cell proliferation differs after culturing the cells for 3 days in either of the three basal media. As shown in Figure 3 at all time points investigated OPCs cultured in DMEM or RPMI showed significantly higher percentages of cells that had incorporated BrdU than cells cultured in NB medium, suggesting that the NB medium inhibits OPC proliferation. A further investigation of the percentage of caspase-3 positive, proapoptotic cells as well as cleaved caspase-3 positive cells, that indicate a further advanced step in the process of apoptosis, yielded a higher percentage of labeled cells after 3 days in NB medium in comparison with the other media (Figure 4). This suggests that in NB medium fewer cells proliferate and more cells undergo apoptosis than in the other two media.

### Effects of osmolarity on OPC proliferation and apoptosis

**Figure 5:**
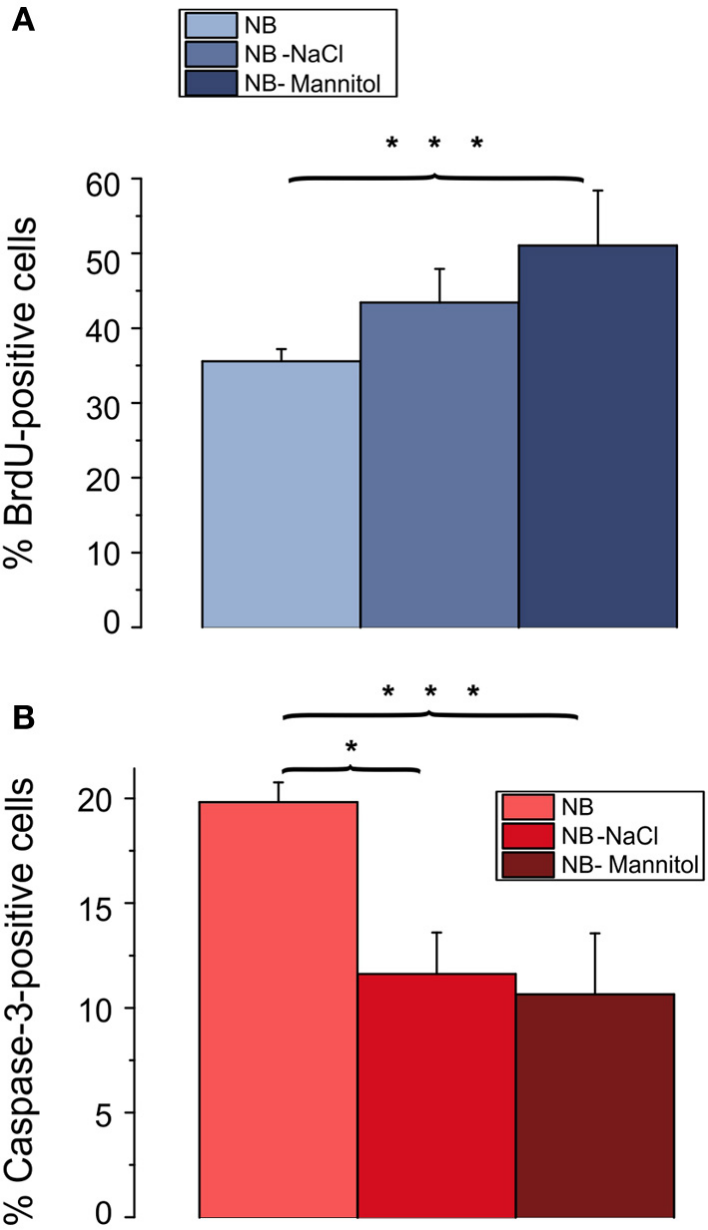
**Effects of osmolarity on percentage of BrdU and caspase-3 positive cells**. Oligodendrocytes maintained for 3 days in NB based culture that had been supplemented with 52.5 mM NaCl or 100 mM mannitol to increase the osmolarity of NB to the osmolarity of DMEM. Note, that a change in osmolarity by either mannitol or NaCl results in an increase in proliferating, BrdU positive cells **(A)** and a decrease in caspase-3 positive, proapoptotic cells **(B)** (4 coverslips from 4 preparations counted, ^*^p < 0.05, ^***^p < 0.005; error bars indicate ± s.e.m., One-Way ANOVA followed by Tukey *post-hoc* Test).

A prominent difference between RPMI, DMEM, and NB is the lower osmolarity of the NB medium. The commercial NB medium used contained only 205 mOsm, compared with 280 mOsm of RPMI and 305 mOsm of DMEM. To test whether the lower osmolarity of NB might have been the cause for the lower proliferation rate and the higher rate of apoptotic cells we increased the osmolarity of the culture medium by increasing the NaCl concentration by 52.5 mM. As shown in Figure 5 this procedure resulted in an increase in the percentage of BrdU incorporating cells (from 36 ± 2% in NB to 43 ± 4% in NB supplemented by NaCl) as well as in a decrease in the percentage of caspase-3 positive cells [from 20 ± 1% in NB to 12 ± 2% in NB supplemented with NaCl (*p* < 0.05), *n* = 4 preparations investigated]. The increase in the proliferation rate of the OPCs could potentially be explained by a larger amino acid uptake through Na^+^-coupled transporters in an extracellular solution containing a larger concentration of Na-ions and thus a larger driving force for Na^+^ influx into the cells. To test, whether this explanation is feasible we increased the extracellular osmolarity of the NB medium by adding 100 mM mannitol. This procedure led to an even larger increase in the percentage of cells showing BrdU incorporation (to 51 ± 7% in NB supplemented by mannitol, *p* < 0.005) as well as to a slightly larger decrease in the percentage of caspase-3 positive cells to 10 ± 3% in NB supplemented with mannitol, *p* < 0.005, *n* = 4 preparations investigated (Figure 5). Hence we conclude, that the lower osmolarity of the NB medium inhibits proliferation and favors apoptosis of OPCs.

### Influence of cell density on differentiation of oligodendrocytes

**Figure 6:**
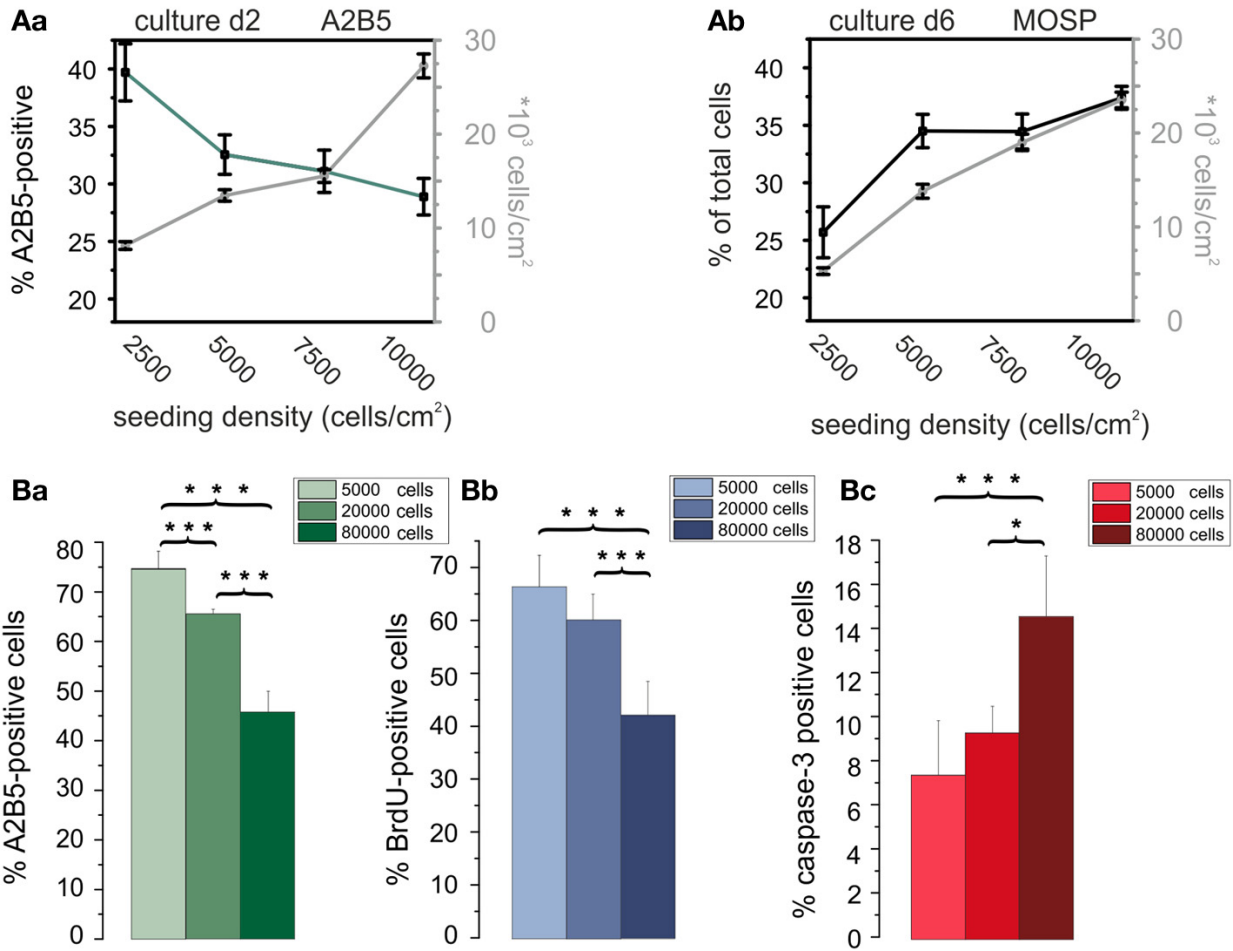
**Influence of cell density on differentiation, proliferation, and apoptosis of OPCs**. **(Aa)** Cell density (data connected by gray lines) and percentage of A2B5-positive cells (data connected by green line) vs. plating density at day 2 in culture. Note, that the percentage of immature OPCs negatively correlates with cell density. **(Ab)** Total cell density (gray line) and percentage of MOSP-positive cells (black line) vs. cell density after 6 days in differentiation medium. Note the larger percentage of differentiated cells in higher plating density. Data shown in **(A)** obtained from RPMI based cultures. Data shown in every bar collected from *n* = 100 frames from 5 preparations. Data for A2B5 as well as MOSP staining between first and last plating density significantly different with *p* < 0.005. **(Ba)** Percentage of A2B5 positive OPCs from a second series of experiments, confirming a higher yield of immature cells in cultures with a larger plating density. **(Bb)** Percentage of BrdU positive cells in dependence of plating density. Note that a higher plating density decreases proliferation rate. **(Bc)** Influence of plating density on percentage of caspase-3 positve cells. Note, that the larger plating density increases the percentage of proapoptotic cells. Experiments shown in **(B)** performed after 3 days of culture in DMEM based medium [2 coverslips counted from 4 preparations (^*^p < 0.05, ^***^p < 0.005; error bars indicate ± s.e.m.)].

To determine optimal cell seeding densities for maximal proliferation rates *in vitro* we investigated potential effects of the cell density on the further development of OPCs. Cells were seeded at four densities (2500, 5000, 7500, and 10000 cells/cm^2^). Then OPCs were incubated either for 2 days in PM on the basis of RPMI to examine the percentage of A2B5-positive OPCs in dependence of cell density or for 6 days in RPMI based differentiation medium to investigate potential cell density effects on the percentage of MOSP-positive differentiated oligodendrocytes. With increasing cell density the percentage of A2B5-positive cells decreased (see Figure 6Aa). After 2 days in PM 40 ± 3% of the cells in the culture were A2B5-positive in the lowest density culture examined. The percentage of A2B5-positive cells decreased with increasing cell density showing the lowest percentage of 29 ± 2% (40 ± 3% to 29 ± 2%, *p* < 0.005) at a cell density of 10,000 cells/cm^2^. This indicates that in lower density cell cultures either more cells proliferate or fewer cells differentiate or undergo apoptosis. In case of a significant increase in proliferation the ratio of seeded to surviving cells should be larger in the lower density cultures. Indeed, the ratio of the number of cells counted after 2 days to the number of cells seeded was 6% higher, amounting 33% in low density cultures (2500 cells/cm^2^) compared with 27% in high density cultures. The percentage of A2B5-positive cells was even 12% higher in the low density cultures, suggesting that the higher cell density indeed induces differentiation of OPCs. To further investigate whether the higher cell density exerts a differentiating effect, OPCs were seeded at densities ranging from 2500 to 10,000 cells/cm^2^ and directly cultured in differentiation medium for 6 days. At d6 in differentiation medium the percentage of MOSP-positive cells varied from 26 ± 2% (2500 cell/cm^2^) to 38 ± 1% (10000 cells/cm^2^) (*p* < 0.005). As illustrated in Figure 6Ab our results indicate that an increasing cell density promotes maturation at the expense of the proliferating A2B5-positive phenotype.

Since the cell density could influence OPC proliferation and/or apoptosis we performed an additional series of experiments in DMEM based PM (see Figure 6A) at a later stage of the experiments to further substantiate the initial findings of the influence of cell density in RPMI based medium, also expanding the range of cell densities studied to higher plating densities (Figure 6B). Immunostaining for A2B5 confirmed, that in DMEM based medium an increase in plating density leads to a decreased percentage of A2B5 positive progenitor cells as well (Figure 6Ba). The increased plating density led to a parallel significant decrease in the percentage of BrdU positive proliferating cells (Figure 6Bb) indicating that the enhanced cell density inhibits proliferation. Furthermore, a significant increase in the percentage of caspase-3 positive proapoptotic cells was observed, indicating that an increase in plating cell density also leads to an enhanced apoptosis rate in the cultures.

### Effects of conditioned media on percentage of A2B5 positive, proliferating, and proapoptotic cells

**Figure 7:**
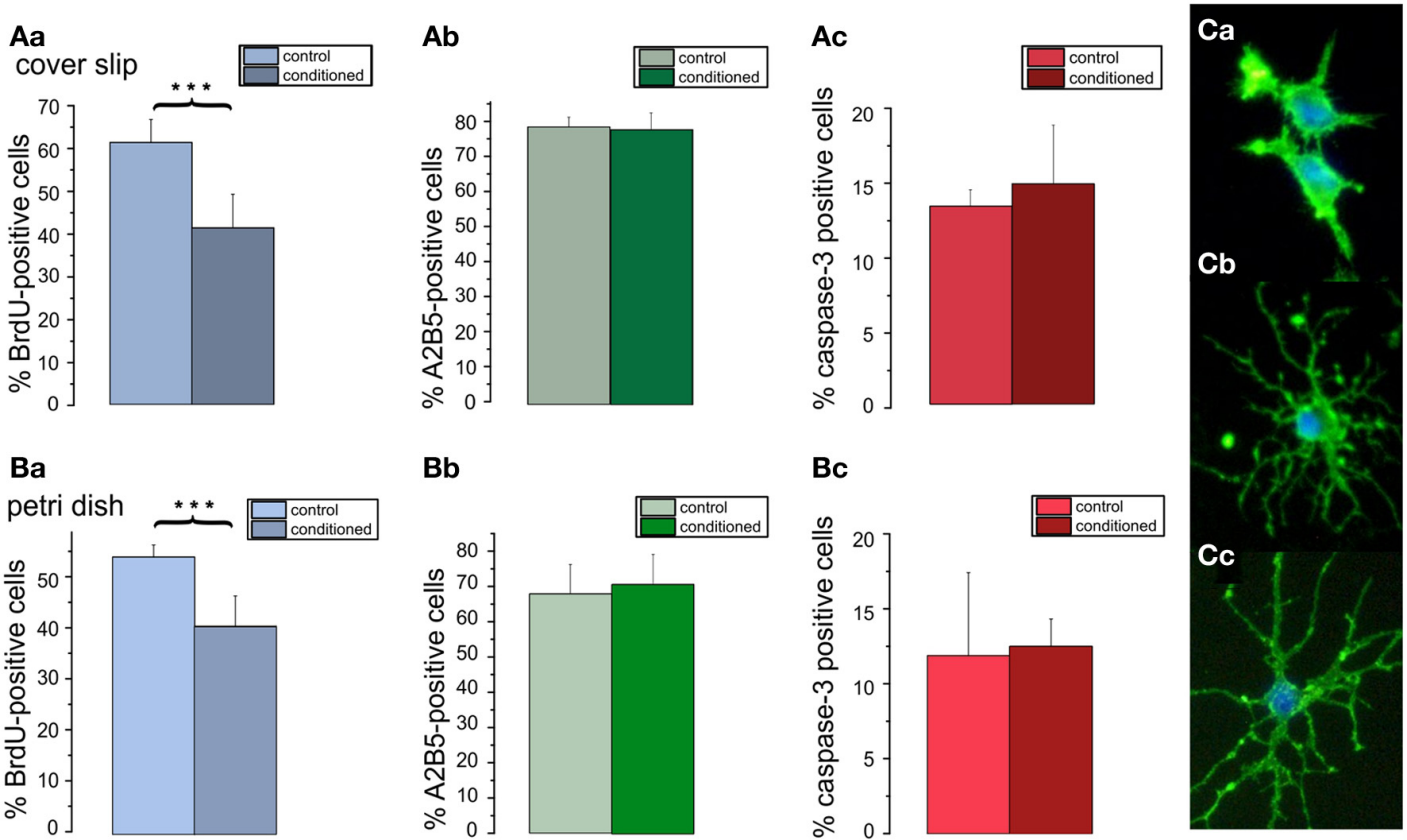
**Effects of conditioned media on percentage of A2B5 positive cells, proliferation, and apoptosis in low density cultures**. Cells plated with a seeding density of 5000 cells/coverslip cultured in DMEM based proliferation medium for 3 days (control) or in parallel with the supernatant of high density cultures (80000 cells/coverslip) (conditioned). Bar graphs shown in **(A)** represent results of immunostainings for **(Aa)** BrdU, **(Ab)** A2B5 **(Ac)** caspase-3 performed on of 5 coverslips from 3 preparations. Bar graphs shown in **(B)** represent results from analogous stainings performed in Petri dishes, both after 3 days in proliferation media. Note, that the conditioned medium decreases the proliferation rate of the cells (^***^p < 0.005; error bars indicate ± s.e.m.). **(C)** A5B5 immunostainings of OPC from control cultures show a less mature morphology than those from conditioned cultures **(Cb,Cc)**.

To investigate, whether soluble cell secreted factors might be the cause for the increased differentiation as well as increased apoptosis of the OPCs in higher density cultures, OPCs were first seeded at a high density of 80,000 cells/cm^2^ in DMEM based medium. After 3 days in culture the medium was removed and added to low density cultures containing 5000 cells/cm^2^ on cover slips or seeded into the center of Petri dishes. Counts of cells immunopositive for BrdU (Figures 7Aa,Ba), A2B5 (Figures 7Ab,Bb), and caspase-3 (Figures 7Ac,Bc) performed after 3 days in PM showed, that the conditioned medium selectively inhibited the proliferation of the OPCs. Although the percentage of A2B5 positive cells was not significantly affected, the conditioned medium exerted a differentiating effect, as evident by a morphologically more mature appearance of the A2B5 positive cells (Figure 7C). The factors contained in the conditioned medium however, did not change the percentage of caspase-3 positive preapoptotic cells. No obvious differences were observed for OPCs seeded on cover slips or in Petri dishes, suggesting that the higher dilution of the secreted factors in the larger fluid volumes of the Petri dishes did not influence the results.

### Effects of surplus microglia on percentage of A2B5 positive, proliferating, and proapoptotic cells

**Figure 8:**
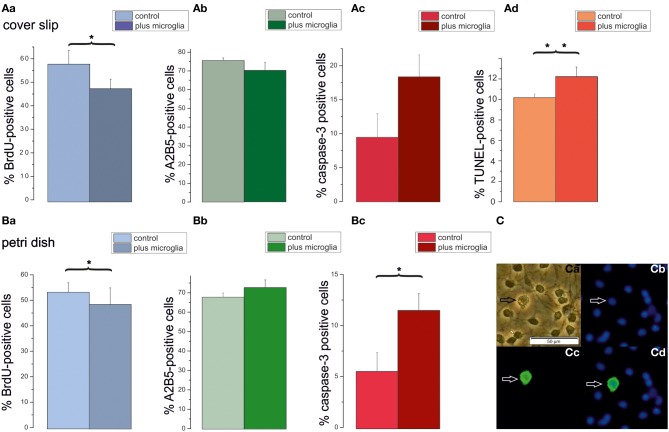
**Impact of additional microglia on OPCs cultured on cover slips or petri dishes**. Cells plated with a seeding density of 5000 cells/coverslip were cultured in DMEM based proliferation medium for 3 days (control) or co-cultivated with an added density of microglia (plus microglia), as counted in high density cultures (80,000 cells/coverslip) in proliferation medium. Bar graphs **(Aa–Ad)** display percentages of OPCs cultured on coverslips. **(Aa)** BrdU-positve cells. **(Ab**) A2B5-positive cells. **(Ac)**: Caspase-3-positive cells, **(Ad)** TUNEL positive cells, 6 cover slips from 3 preparations investigated. Bar graphs in **(Ba–Bc)** display percentages of OPCs cultured on Petri dishes. **(Ba)**: BrdU-positive cells. **(Bb)** A2B5-positive cells. **(Bc)** Caspase-3-positive cells; 6 cover slips from 3 preparations investigated. Error bars indicate ± s.e.m. ^*^p < 0.05, ^**^p < 0.01; ANOVA followed by Tukey *post-hoc* Test. **(C)** Examples of OX-42 stained microglia after 3 days in DMEM based proliferation medium, seeding density 80,000 cells/coverslip **(Ca)** phase contrast picture, **(Cb)** Hoechst 33258 staining of the same image, **(Cc)** OX-42 immunofluorescence, **(Cd)** merged pictures, calibration bar indicates 50 μm.

The preceding results suggest that secreted factors from neighboring OPCs inhibit proliferation and induce differentiation. However, the impact of the high plating density on caspase-3 positive cells could not be explained by these experiments. Since a larger plating density also leads to more microglia in the respective cultures we performed an additional series of experiments in which additional microglia were added to low density OPC cultures. For these experiments the number of microglia per coverslip, respectively Petri dish, was determined from OX-42 stained cultures. Cultures seeded at a density of 80,000 cells/coverslip contained a low percentage of microglia (3.6 ± 0.2%, *n* = 4 preparations), which amounted to 2700 cells per coverslip. Cultures seeded at a low density of 5000 cells/coverslip were co-cultivated with an additional number of 2700 microglia, amounting to nearly a microglia to OPC ratio of 1:2. As shown in Figure 8 the presence of additional microglia significantly inhibited BrdU incorporation, albeit to a smaller extent than the conditioned medium. In contrast to the conditioned medium, the extra dosage of microglia significantly increased the percentage of caspase-3 positive proapoptotic OPCs. This finding was confirmed with a TUNEL assay on coverslip cultures, showing a similar percentage of apoptotic cells. These results show that the microglia present in the higher density cultures could explain the higher rates of proapoptotic cells observed in high density cultures.

### Effects of cell density on cytokine-effects on the populations of A2B5- and MOSP-positive cells

Cultures offer the advantage that cell types can be purified and this makes it possible to study drug actions on cell populations of varying composition, and to work out interactions between different cell types in a way that is not possible *in vivo*.

**Figure 9:**
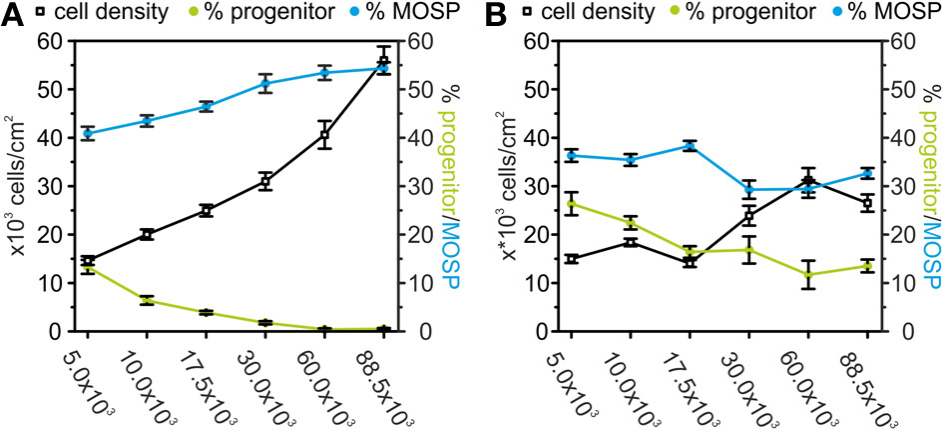
**Effects of TNF-α and IFN-γ treatment on A2B5-positive progenitors and MOSP-positive oligodendrocytes after 9 days in serum-free culture. (A)** Dependence of the number of surviving cells (counted from DAPI-positive nuclei—black), progenitor cells (green), and MOSP-positive oligodendrocytes (blue) after 3 days of incubation in proliferation medium and 6 days in differentiation medium based on RPMI. Note the decrease in the percentage of bipolar progenitor cells and increase of MOSP-positive cells with increased cell density at time of seeding. **(B)** Dependence of the number of surviving cells (counted from DAPI-positive nuclei—black), bipolar progenitor cells (green), and MOSP-positive oligodendrocytes (blue) under identical conditions as used for data shown in **(A)** with the exception of adding 10 ng/ml TNF-α and 20 ng/ml IFN-γ from d2–d3. Note the increase in the percentage of progenitor cells and the decrease in the percentage of MOSP-positive cells at all cell densities studied. Error bars indicate ± s.e.m.

We have previously described an inhibition of OPC survival and differentiation after 2 days of treatment of proliferating OPCs in culture with the cytokines TNF-α and IFN-γ (Feldhaus et al., 2004a; Mann et al., 2008). Since the results shown above indicated that the percentage of A2B5 positive cells in the OPC culture depends to some extent on cell density and basal medium we here repeated our previous treatment protocol of OPCs using different plating densities in RPMI based proliferation and differentiation medium. Cells were maintained for 3 days in PM, the last two of which included cytokine-treatment, followed by a differentiation phase of further 6 days in cytokine-free differentiation medium. As shown in Figure 9 the cell density was larger in cultures with a high seeding density. However, as already observed in Figure 6A, the increase in surviving cells was smaller than the increase in plating density. Here an increase in seeding density by a factor of 17 only increased the yield of cells by a factor of 4. Furthermore, in accordance with our observations shown in Figure 6Aa, the percentage of progenitor cells declined with increasing number of cells seeded from 13.5% at the lowest cell density to less than 0.7% at the highest seeding density. In further agreement with the results shown in Figure 6Ab the percentage of MOSP-positive cells increased in the higher density cultures.

In further agreement with previous results (Feldhaus et al., 2004a) a cytokine-treatment of the proliferating cells for 48 h induced a significant decrease in the number of the surviving cells. At all densities investigated, the percentage of the MOSP-positive cells was significantly lower and the percentage of progenitor cells was significantly higher after 6 days in differentiation medium following cytokine-treatment. The cytokine-effect on cell survival as well as block of differentiation was largest in the highest density culture. However, in the lowest density culture the cytokine-treatment had no effect on cell number, but still significantly increased the percentage of progenitor cells (*p* < 0.005) and decreased the percentage of MOSP-positive cells (*p* < 0.05) (Figure 9B). The stronger effect of the cytokine treatment in the high density cultures could be explained by the small percentage of about 5% microglia in the culture which reach higher absolute numbers in the high density cultures. Since the cytokine treatment could have additionally activated microglia to secrete further cytokines, the effect of the cytokine treatment might have been amplified by the residual microglia in the high density cultures.

## Discussion

### Differences in basal media

The main novel result of the present investigation is that the Neurobasal based culture medium strongly reduces the proliferation of OPCs and enhances apoptosis. After a preculture period in PM and an additional incubation in NB-based differentiation medium the number of A2B5-positive cells was reduced to less than 3%. In contrast, when the cultures were incubated with identical serum free medium supplements on the basis of RPMI, the number of cells in the culture continued to increase during the first week leading to a much higher yield of surviving and of differentiated MOSP positive cells. The higher yield of cells in the cultures could be explained by the continued maintenance of a proliferating progenitor cell pool as evidenced by the higher rate of BrdU positive cells in cultures maintained in DMEM or RPMI in addition to a decrease in apoptotic cells as evidenced from the decreased percentage of caspase-3 positive cells in the latter media. Hence, if a culture with a minimal percentage of A2B5-positive cells after differentiation is required, NB medium is the basal medium of choice for studying differentiated oligodendrocytes. If a larger absolute number of surviving cells is required, basal media such as RPMI or DMEM are recommended.

Since NB differs from RPMI as well as DMEM by a strong difference in osmolarity we investigated whether adding NaCl or mannitol to the culture medium could influence BrdU incorporation as well as Caspase-3 immunopositivity. In a first series of experiments we found that the addition of NaCl strongly increased the percentage of BrdU positive cells to near the values found in DMEM (data not shown). In a second series of experiments we studied, whether this effect was due to the reduced Na^+^ concentration in the NB medium or induced by changes in osmolarity. Our results showed that the BrdU incorporation rate showed an even stronger increase and the percentage of caspase3-positive cells was even lower if mannitol was added to the culture medium, suggesting that an increase in osmotic pressure increases OPC proliferation. Although the inventors of the Neurobasal medium noticed a reduction of gliosis in the culture they did not investigate in more detail the effect of reducing the osmotic pressure on the proliferation of oligodendrocytes (Brewer and Cotman, 1989; Brewer et al., 1993). Although to our knowledge this effect has not been noticed before on oligodendrocyte lineage cells, increases in cell volume have been observed in proliferating fibroblasts, mesangial cells, lymphocytes, HL-60 cells, GAP A3 hybridoma cells, smooth muscle cells, and HeLa cells (cited in Lang et al., 1998). However, in most of these cell types, cell swelling has been associated with proliferation whereas cell shrinkage rather correlated with apoptosis (Lang et al., 1998). This is somewhat in contrast to the present findings, since the hypoosmolar NB medium should at first induce a cell swelling. Potentially, our results could be explained by an overcompensation of the transient short term osmotic effects during a culture period of several days. Alternatively, in hypoosmolar solutions, cyclic volume changes might be reduced, resulting in an inhibition of ion fluxes required for cell proliferation, such as volume activated anion channels (see e.g., He et al., 2012).

### Further factors influencing survival and proliferation of OPCs

Our results, showing that OPCs proliferate better in DMEM compared with NB, are in contrast to the observations of Yang et al. who showed that B27/NB medium was significantly more effective in maintaining viable cells and in supporting oligodendrocyte proliferation than the combination N1/DMEM (Yang et al., 2005). Since our present results provide strong evidence that the lower osmolarity of NB inhibits proliferation, we suggest, that the additional ingredients in B27 compared with N1 obviously overcome the inhibitory effect of NB, in sum resulting in the enhanced proliferation observed by Yang et al., 2005.

In addition to the factors contained in B27 (Brewer et al., 1993) or NS21 (Chen et al., 2008), further factors have been shown to promote OPC proliferation, such as the supplement of PDGF (Noble et al., 1988; Raff et al., 1988; Pringle et al., 1989; Barres et al., 1992) as well as FGF-2 (Bogler et al., 1990; McKinnon et al., 1991; Grinspan et al., 1993) used here to induce OPC proliferation. These factors not only play a role in OPC proliferation *in vitro* but have been shown to be involved in OPC proliferation *in vivo* as well. The current concept is that FGF functions downstream of sonic hedgehog (Shh) which induces ventrally-derived OPCs (Nery et al., 2001) and acts predominantly via the FGF receptors FGFR1 und FGFR2 to supply OPCs *in vivo* (Furusho et al., 2011). Most prominently, FGFR1 and FGFR2 seem to be required *in vivo* to assemble the normal number of myelin wraps around axons in the course of maturation (Furusho et al., 2012) using the ERK1/2- MAPK downstream signaling cascade (Ishii et al., 2012). Activation of ERK1/2 is, however, not only responsible for extensive myelination but also essential in the earlier step of OPC generation (Ishii et al., 2013). In accordance with the prominent role of FGF-2 *in vivo* is the finding of enhanced FGF-2 levels in multiple sclerosis lesions, which could originate from activated astrocytes and microglia and help to repopulate the tissue with OPCs (Clemente et al., 2011).

Besides adding PDGF and FGF-2 to the PM alternative methods have been described to further stimulate OPC proliferation in culture. Hence it has been shown that conditioned medium from B104 neuroblastoma cells stimulates OPC proliferation by secretion of a PDGF AA and AB dimer independent factor, which could be especially useful for expanding adult brain derived OPCs (Hunter and Bottenstein, 1991). Conditioned medium from B104 neuroblastoma cells was also successfully used to induce OPC proliferation on astrocyte feeder layers and increase the yield of isolated OPCs (Niu et al., 2012) as well as to induce OPCs from embryonic mouse neurosphere cultures (Chen et al., 2007). However, other authors came to the conclusion, that the combination of FGF-2 and PDGF is more efficient than the combination of B104 conditioned medium with FGF-2 to expand OPCs from E16 rat spinal cord (Fu et al., 2007). Further antioxidants could exert protective effects on cultured OPCs (Cammer, 2002) and additional regulatory factors, which are released *in vivo* for e.g., from axons (Barres and Raff, 1999) continue to be identified (Sher et al., 2008). Such potential factors include hepatocyte growth factor (HGF) (Yan and Rivkees, 2002), which potentiates the proliferation enhancing effect of heregulin (HRG), a glial growth factor (Canoll et al., 1996). Further factors, such as brain derived neurotrophic factor (BDNF) (Vondran et al., 2010), neurotrophin-3 (NT-3) (Kahn et al., 1999) as well as insulin-like growth factor type 1 (IGF-1) (Zeger et al., 2007) have been identified as factors promoting proliferation of oligodendrocytes. Furthermore, it would be useful to test, whether the addition of Shh which increases OPC proliferation (Merchán et al., 2007; Wang et al., 2008) could additionally increase the yield of OPCs in culture. In order to further optimize culture media yielding maximal numbers of OPCs in addition to PDGF and FGF-2 further factors will have to be added to the PM.

### Effects of cell density on maturation of oligodendrocytes

Additional factors secreted paracrinely from OPCs could also explain our second observation, that the density of the cells at the time of seeding had a clear influence on proliferation and differentiation in culture. Thus, the percentage of A2B5-positive cells decreased already under conditions favoring proliferation, and the percentage of differentiated MOSP-positive cells increased after incubation in differentiation promoting solution with increasing density of oligodendroglial cells. The influence of the conditioned medium on BrdU incorporation showed that OPCs secrete factors that inhibit proliferation and promote differentiation. Furthermore cell death in very low density cultures suggests that the cells require additional paracrinely secreted survival signals not present in sufficient concentration in our serum replacement formulation. These findings are in agreement with results by Levi and Agresti (1991) who found that O-2A progenitors cultured at high densities largely differentiated into mature oligodendrocytes. Furthermore they suggested that O-2A progenitors secrete high molecular weight, non-mitogenic factors capable of inducing a rapid differentiation (Levi et al., 1991). Such autocrinely or paracrinely released factors, influencing survival and differentiation of OPCs could e.g., include endocannabinoids (Molina-Holgado et al., 2002), as well as TGF-β which could promote differentiation (McKinnon et al., 1993).

### Effects of microglia on OPCs

We observed that the addition of microglia to the cultures resulted in a weak inhibition of BrdU incorporation and a strong increase in the percentage of caspase-3 expressing preapoptotic and TUNEL-stained apoptotic cells. This is surprising, since microglia secreted factors should have also been present in the conditioned medium, which only influenced proliferation and not apoptosis. This discrepancy could be explained by the fact, that OPCs could secrete TGF-β (McKinnon et al., 1993), which in turn has been shown to inactivate microglial cells (Spittau et al., 2013). Our results confirm previous observations that activated microglia may act deleterious to OPCs (Miller et al., 2007; Pang et al., 2010). Likewise, Taylor et al. (2010) observed an inhibition of proliferation by microglia and identified interleukin-6 and tumor necrosis factors (TNFs) as potential factors involved. Selmaj et al. (1991) additionally identified TNF-α and IFN-γ as factors released from microglia and astrocytes and (Agresti et al., 1996) showed that these factors inhibit proliferation of OPCs.

### Cytokine-effects at different cell densities

A major disease effecting OPCs in neurodevelopment is periventricular leukomalacia (PVL), which can lead to spastic palsy and mental retardation (Dammann and Leviton, 1997; Benarroch, 2009). PVL can be caused by infection and inflammation or by hypoxic-ischemic episodes (Berger and Garnier, 1999; Du Plessis and Volpe, 2002; Ellison et al., 2005) and is therefore considered to be of multifactorial origin. Increased numbers of reactive INF-γ positive astrocytes (Folkerth et al., 2004) as well as increased levels of TNF-α, have been found in autopsies of children with PVL (Kadhim et al., 2001), suggesting that these cytokines could contribute to the generation of this disease. Studies performed on cultured purified oligodendrocyte progenitor cells provided evidence that the exposure of OPCs to INF-γ and TNF-α in fact induces cell death and additionally prevents the morphological differentiation of oligodendrocyte progenitors as well as the expression of myelin-specific proteins (Agresti et al., 1996; Andrews et al., 1998; Cammer and Zhang, 1999; Feldhaus et al., 2004a; Chew et al., 2005) confirming the concept that these factors could possibly be involved in the generation of PVL. In addition, further cytokines, like IL-1β, which are released as well as act on oligodendrocytes (Blasi et al., 1999; Vela et al., 2002) have also been shown to cause PVL like symptoms and axonal damage after systemic administration (Favrais et al., 2011).

Previous evidence indicates that the treatment with INF-γ and TNF-α not only impairs survival but leads to a larger population of undifferentiated cells than observed in control cultures after one week of differentiation (Cammer and Zhang, 1999; Feldhaus et al., 2004a). Cytokine-treated cultured oligodendrocytes express less myelin proteins at the mRNA (Feldhaus et al., 2004b) as well as the protein level and maintain their immature complement of voltage-gated ion channels (Mann et al., 2008). Furthermore after OPC transplantation into lipopolysaccharide-lesioned rat brain tissue the transplanted OLs show a reduced differentiation (Webber et al., 2009), which could be explained by cytokine actions, as observed in culture.

On might argue, that the impaired differentiation observed in cell culture experiments stems from the dilution of the differentiation promoting factors secreted at high cell densities (see Figures 6, 7), which would increase with an increasing cytokine induced cell death in the culture. The impairment of differentiation observed after cytokine treatment would then be an indirect effect caused secondarily to the reduction in cell density. To learn more about potential cell density effects on the cytokine actions we thus performed a 48 h treatment with 10 ng/ml TNF-α and 20 ng/ml IFN-γ in cultures seeded at different cell densities (Figure 9). Our results show, that even at the lowest plating density, at which no cytokine effects on the number of surviving cells was observed, the cytokine treatment increased the percentage of immature cells and decreased the percentage of differentiated cells. This suggests, that combined treatment with INF-γ and TNF-α directly inhibits differentiation of OPCs.

Most interestingly, the increase in the number of bipolar progenitor cells and the reduction of the percentage of surviving cells as well as of MOSP-positive differentiated cells in response to the cytokine treatment was more pronounced in high density cultures than in low density cultures. In accordance with the results of (Hewett et al., 1999; Pang et al., 2010) this effect could be explained by the larger absolute number of microglia cells in the high density cultures, which could be stimulated by the cytokines to release further deleterious cytokines thus amplifying the cytokine effects on OPC survival and differentiation.

## Conclusions

In order to work out concepts for myelin repair strategies to obtain high yields of cultured OPCs are desirable. These cells can then be used to perform pharmacological as well as biochemical investigations to investigate the properties of purified OPCs in isolation. Furthermore, purified OPC cultures could potentially be useful for transplantation experiments, as already performed by Bambakidis and Miller (2004) and Webber et al. (2009). Further differentiation of transplanted OPCs *in vivo* might, however, be compromised by signals from the native environment, preventing the further differentiation into myelinating oligodendrocytes (Lü et al., 2010; Sypecka and Sarnowska, 2013). It will have to be worked out in future, whether the addition of further factors (see e.g., Bambakidis and Miller, 2004) could potentially help to restore functional myelination.

To obtain satisfactory numbers of OPCs *in vitro* adequate culture conditions have to be worked out. The first step would be to start with a high number of purified OPCs. To obtain purer cultures immunopanning (Barres et al., 1992), generation from neurospheres (Itoh, 2002; Pedraza et al., 2008), which can also be used to obtain OPCs from mice (Chen et al., 2007) and Magnetic Activated Cell Sorting (MACS) (Dincman et al., 2012) methods have been developed which, however reduce the yield of cells. An interesting alternative, leading to much higher numbers of OPCs to start with and causing lower costs is a recently developed expansion of OPC growth on an astrocyte feeder layer with neuroblastoma cell conditioned media (Niu et al., 2012).

In addition to expanding cells with proliferation promoting factors such as PDFG and FGF-2 which were used in the present experiments, further factors, such as HGF (Yan and Rivkees, 2002), HRG (Canoll et al., 1996) or IGF-1 (Zeger et al., 2007) or Shh (Gao, 2006; Merchán et al., 2007) wait to be tested in order to clarify whether a different combination of growth factors could further increase the yield of OPCs. Here, we worked out, that, additionally, the basal culture medium has a significant influence on proliferation and apoptosis of OPCs. Particularly we observed, that a decrease in the osmolarity of the medium reduces proliferation and increases apoptosis of OPCs. If a culture of differentiated oligodendrocytes with a low number of remaining OPCs is desired, the low osmolarity Neurobasal medium would be the medium of choice. If a high yield of OPCs is required a medium with higher osmolarity, favoring proliferation, such as DMEM or RPMI is recommended.

Furthermore, the experiments shown here confirm that oligodendrocyte lineage cells secrete differentiation promoting factors. If a high proliferation rate is desired cells should be maintained at cell densities of about 5000 cells/cm^2^.

Since cells secrete paracrinely acting factors some pharmacological effects observed may to some extent depend on the cell density in the cultures. This was demonstrated here by a series of experiments showing that cytokines exert stronger effects on survival and differentiation of oligodendrocyte lineage cells in higher than in lower density cultures. This can be explained by the activation of a small percentage of 3–5% residual microglial cells, amplifying the cytokine effects by releasing additional cytokines upon activation.

Taken together, the highest yield of oligodendrocytes is obtained in media of high osmolarity, seeded at low density in the presence of a low concentration of microglia. Since microglia have been shown to be not only deleterious but also secrete survival promoting factors (Miller et al., 2007), it has to be worked out, whether it would be beneficial to inactivate, but not totally remove remaining microglia, for instance by supplementing TGF-β (Spittau et al., 2013).

## Author contributions

Karolina Kleinsimlinghaus, Romy Marx, Meray Serdar, Ivo Bendix, and Irmgard D. Dietzel conceived and designed the experiments, which were carried out by Karolina Kleinsimlinghaus, Romy Marx, and Meray Serdar assisted by the undergraduate students listed in the Acknowledgement. Karolina Kleinsimlinghaus, Romy Marx, and Irmgard D. Dietzel predominantly wrote the manuscript. All authors have read the manuscript before submission.

### Conflict of interest statement

The authors declare that the research was conducted in the absence of any commercial or financial relationships that could be construed as a potential conflict of interest.
